# The involvement matrix as a framework for involving youth with severe communication disabilities in developing health education materials

**DOI:** 10.1111/hex.13445

**Published:** 2022-02-11

**Authors:** Shakila Dada, Adele May, Kirsty Bastable, Alecia Samuels, Kerstin Tönsing, Jenny Wilder, Maureen Casey, Constance Ntuli, Vasu Reddy

**Affiliations:** ^1^ The Centre for Augmentative and Alternative Communication University of Pretoria Pretoria South Africa; ^2^ Stockholm University Stockholm Sweden; ^3^ Department of Sociology University of Pretoria Pretoria South Africa

**Keywords:** augmentative and alternative communication, COVID‐19 pandemic, involvement, Involvement Matrix, severe communication disabilities, youth, engagement

## Abstract

**Introduction:**

Involving youth with severe communication disabilities in health research is foregrounded in a perspective of rights and participation. Researchers aligned with a participatory and inclusive research agenda recommend that involving youth in health research should be a deliberate and well‐planned process. However, limited examples exist of how researchers can facilitate the involvement of youth with severe communication disabilities in research projects.

**Method:**

The aim of this paper was to describe the application of the Involvement Matrix as a conceptual framework to guide the three phases of a research project with youth with severe communication disabilities.

**Results:**

Six youth aged 19–34 years consented to be involved in the project. All youth had a severe communication disability and used augmentative and alternative communication (AAC) to support their involvement in the research project. The Involvement Matrix provided a structure to delineate four involvement roles in three research phases: In Phase 1, youth were *listeners* to research information and *advisors* in the needs analysis. In Phase 2, as *advisors* and *decision‐makers*, youth provided their opinions on selecting picture communication symbols for health materials. In Phase 3, as *partners*, they were copresenters at an online youth forum.

**Conclusion:**

The Involvement Matrix was used to plan and implement the involvement of youth with severe communication disabilities in codeveloping health materials for use during the COVID‐19 pandemic. The Involvement Matrix can be applied together with AAC to enable meaningful involvement of youth in a health research project as listeners, advisors, decision‐makers and partners.

**Patient or Public Contribution:**

This study project was codeveloped with youth with severe communication disabilities who use AAC in South Africa. A person with lived experience was involved as an advisor to the health material development process and in the drafting of the manuscript.

## 1 INTRODUCTION

Involvement of direct stakeholders (the individuals directly impacted) in health research has gained momentum with an international spotlight on participatory and inclusive research agendas.^1^, ^2^, ^3^ These agendas highlight the need for research to be carried out *with* those for whom it is intended and not merely on or for them.^4^ In participatory research, individuals living with a health condition are actively involved in making decisions and providing input into the various phases of the research process.^5^

The inclusion of direct stakeholders in the research process changes the power dynamic as the stakeholders are recognized as collaborative partners due to their intrinsic strengths and experiential knowledge gained through lived experience.^6^ Several models have attempted to categorize the various degrees of participation of stakeholders (especially children and youth) in the research process. The most prominent of these models is Hart's^7^ ladder of participation. The ladder metaphor for participation builds on the seminal work of Arnstein^8^ and represents an upward progression of participation as different rungs on a ladder from varying degrees of nonparticipation up to full participation of stakeholders in projects that are stakeholder led.^6^, ^7^

A critique often levelled at the participation ladder is that it suggests a hierarchy, with the top rung of stakeholder‐initiated research and shared decision‐making being the desired goal.^9^ However, this level of participation may not always be possible or preferred. The level of participation desired and/or possible is dependent on the field, nature, and stage of a research project as well as the capabilities, characteristics, and needs of stakeholders.^9^ In the field of participatory design, a more reflexive approach and nuanced understanding of how control is shared between researchers and participants should be considered.^10^

This development is of importance for a variety of stakeholders. However, it is of utmost pertinence for youth with severe disabilities, including youth who use augmentative and alternative communication (AAC) to overcome severe communication disabilities.^11^, ^12^ Youth with severe communication disabilities are a vulnerable group of the population, whose opinions have generally been excluded from research.^11^, ^12^, ^13^ Moreover, when they are consulted about their opinions, they are usually recruited as research participants on whom research is conducted.^12^, ^14^ Rarely are they brought to the centre of the research process where they are valued for their expertise and their involvement in health research.^15^, ^16^, ^17^ Three factors may contribute to their exclusion in health research involvement:

First, severe disabilities manifest during the developmental years, and may be expressed as permanent, life‐long intellectual disability with associated physical impairment (e.g., quadriplegia), sensory impairment (e.g., loss of vision) or other chronic health conditions (e.g., epilepsy).^18^, ^19^ Due to the presence of developmental disabilities, researchers may have negative assumptions of the capability of youth with severe disabilities to be involved in research in a meaningful way.^20^, ^21^

Second, their communication difficulties typically result in complex communication needs.^22^ The complexity of communication challenges in comprehension and expression often requires the use of AAC. AAC may include electronic devices (e.g., speech‐generating devices), tools (e.g., picture communication symbols on paper‐based boards), or strategies (e.g., gestures, head nods) to involve youth with severe communication disabilities to participate more productively in expressing their healthcare needs.^22^, ^23^ Because youth who use AAC require a greater level of communication support to facilitate their involvement in research, researchers may perceive this as requiring extra time and effort.^17^, ^24^

Third, despite research highlighting the ability of youth to provide input based on personal perspectives and lived experience,^25^, ^26^, ^27^ youth, in general, are often viewed from a deficit‐based perspective as risky, problematic,^28^, ^29^ and lacking in capacity.^30^ In sum, this means that youth with severe communication disabilities face greater barriers to participation in research than their typically developing peers^11^, ^17^ and are often under‐valued by researchers for their role in research.^21^

However, from a perspective of rights and participation, efforts should be made to enhance their involvement in health research.^31^, ^32^ The United Nations Convention on the Rights of the Child (UNCRC), through Article 12, asserts the fundamental right of children and youth to voice their opinion in matters that affect them in accordance with their age and maturity.^33^ Furthermore, Article 23 of the UNCRC (1989) mandates the creation of conditions to facilitate their participation and involvement in daily life.

Implicit to these rights is the acknowledgement of their agency to express themselves, to have autonomy over their bodies, and to engage in decision‐making over their healthcare needs.^33^ In fact, health interventions such as health‐related materials are more likely to be relevant and impactful when youth with severe communication disabilities are involved in the development process with researchers.^17^, ^34^

Involvement in health interventions has escalated in significance under the current global COVID‐19 pandemic. Youth with severe communication disabilities require access to inclusive health education not only to understand newly adjusted health and social protocols but also to express their health needs as COVID‐19 symptoms arise.^35^ People with disabilities are generally a vulnerable group in terms of COVID‐19. They are at higher risk for contracting the Coronavirus as they often have to rely on caregivers or live in residential facilities.^36^ Given the imperative to tailor inclusive health‐related COVID‐19 information to accommodate the cognitive, sensory and physical needs of youth with severe communication disabilities,^35^ creating opportunities for their involvement in health research is an urgent priority.^37^

To enable this type of research involvement, two actions are required. On the one hand, it is incumbent on researchers to believe that youth with severe communication disabilities play a unique role in shaping the development of health interventions.^38^ On the other hand, youth with severe disabilities need to be given clarity on their roles and explanations of how these roles will be enacted alongside researchers.^38^ Researchers therefore have a responsibility to execute these actions in a thoughtful and well‐planned manner.

However, it has been shown that researchers are unsure of how youth with severe communication disabilities can be involved practically in research projects.^39^, ^40^ They may also be unaware of tools that can facilitate their involvement.^16^, ^41^ The Involvement Matrix^40^ is one such tool that can help to facilitate discussion about degrees of involvement and provide clarity about roles and expectations. There has been a call from researchers to share examples and lessons learned from the application of tools that involve youth with disabilities in health research.^42^, ^43^ Therefore, the aim of this paper is to share one example by describing how the Involvement Matrix^40^ was applied in our project as a tool to involve youth who use AAC, in health research.

This paper reports on the application of the Involvement Matrix^40^ to conceptualize and delineate the roles of youth who use AAC in a health research project concerning accessible and appropriate health education materials. The health research project and the Involvement Matrix^40^ are described in Section Method, 2, where information is also provided about the youth using AAC and their involvement in the health research project. The roles that they took up and the significance of these roles are described in Sections Results, 3 and Conclusion, 4.

## 2 METHODS

### 2.1 The current research project

The research project was underpinned by the paradigm of participatory research through involvement of youth who use AAC. Within discourses on participatory research, a broad spectrum of terminology is used to describe various types of involvement in research, for example, coproduction^42^ and codesign.^43^ In the current study, public and patient involvement is defined as ‘research that is carried out *with* those for whom research is intended and not merely on or for them’.^4^ Within the context of this study, we refer to codevelopment as the process of youth with disabilities working with the researchers to develop health educational materials.^34^

The current research project aimed to codevelop accessible health education materials with youth who use AAC for use in the COVID‐19 pandemic in South Africa. The research project was undertaken between August 2020 and December 2020 during the COVID‐19 pandemic in the context of adjusted lockdown restrictions in South Africa. The research project comprised health and disability researchers and included youth with lived experiences of severe disability. The research project was conducted in three research phases: Phase 1 (preparation), Phase 2 (codevelopment), and Phase 3 (dissemination). In the first phase (preparation), a needs analysis was conducted to determine the health education needs of youth who use AAC. This was conducted directly with the youth themselves and indirectly with caregivers of youth with disabilities and professionals who work with youth with disabilities. Only the direct involvement with the youth who use AAC is reported in this article.

In Phase 2 (codevelopment), youth who use AAC were invited to work with the researchers to undertake a process of codeveloping health education materials. In Phase 3 (dissemination), the information related to Phases 1 and 2 was shared at an online youth forum copresented by youth and a member of the research team. The perspectives of youth, health professionals and caregivers (Phase 1) and specific details of the health education materials (Phase 2) do not fall within the scope of the current paper and will be described in forthcoming publications by the researchers.

The outcome of the research project was a set of codeveloped materials related to various health topics (e.g., pain, general healthcare and communication about COVID‐19). These health education materials were created in inclusive and accessible formats for youth who use AAC. These formats included picture communication symbols on communication boards and social stories in video animation in six South African languages.

The current paper reports on the Involvement Matrix that was applied to describe the involvement of youth who use AAC in the research project. The specific objectives of the current paper are to describe:

1. the process of applying the Involvement Matrix to guide the involvement of youth who use AAC in this study project and
2. the roles that they took on during the three research phases of the project.

### 2.2 Materials: Involvement Matrix

The Involvement Matrix^40^ was recently developed through cocreation by researchers and stakeholders (also called experience experts). It has been suggested to be a useful conversational tool to help researchers and experience experts to concretize and delineate roles and parameters of involvement within a participatory research project.^40^ The Involvement Matrix was developed within the paradigm of participatory research and principles of patient and public involvement (PPI), that is, (i) respecting an individual's right to be involved in research that affects them, and (ii) incorporating their lived experience in the research in a way that complements the expertise of researchers.^40^

The Involvement Matrix^40^ captures three research phases categorized as ‘preparation’, ‘execution’ and ‘implementation’. These research phases are associated with five different roles ranging from a listener (receives information), cothinker (provides opinions), advisor (provides advice), partner (is an equal collaborator in the project), and decision‐maker (makes decisions). An outline of the Involvement Matrix^39^ package can be accessed at https://www.kcrutrecht.nl/involvement-matrix/.

The matrix is formed by combining the research phases with the different roles. The Involvement Matrix^40^ is flexible in its application, which means that it may be applied during the planning stages before a research project begins or after the research project to evaluate and report on the roles executed in the research project. The Involvement Matrix^40^ was recently applied in research with children with chronic physical and mental health conditions.^3^ In the current project, the researchers applied the Involvement Matrix^40^ with the purpose of guiding the planning of how youth who use AAC could be involved in the research project (Phase 1) and to map out the project activities. It was also used to support this involvement in Phases 2 and 3 based on their interest, availability and consent to be involved.

### 2.3 Youth who use AAC

#### 2.3.1 Recruitment

In this study project, youth who use AAC were recruited within the age range of 18–34 years as per South Africa's *Youth Commission Act* (1996). Youth from across the nine provinces in South Africa who had a severe disability were considered eligible to participate in the study. As per the definition of severe disability, youth were recruited based on their lived experience of having a severe communication disability. All youth used AAC systems and required AAC as a mechanism of communication for participation in the study.

Convenience sampling was used to identify and recruit potential youth with severe communication disabilities from a database at the Centre for Augmentative and Alternative Communication. Recruitment of youth took place between July 2020 and August 2020. In Phase 1, an infomation letter was distributed to the potential youth via WhatsApp and email to explain the purpose of the study and their expected role in this phase of the study. Each aspect of information was presented using simple sentences and pictures to aid understanding. This was conducted using Qualtrics^©^,^44^ a cloud‐based platform for creating and distributing online surveys. In addition, the information was presented in an auditory format using the audio function on Qualtrics^©^ ^44^ to enable participants with lower literacy levels to listen to the questions being read. The participant opened the questionnaire and it automatically began reading the first piece of information to them. Once the information had been presented, the participants were asked to indicate if they understood the information. If they responded ‘yes’ (through selecting an option, which was also described in the auditory feedback), they were moved onto the next piece of information. If the participants indicated that they did not understand the information, then the consent process was stopped and a follow‐up online interview was conducted. Once all the information had been presented, each aspect of consent was recast and the participants were asked to consent to be a part of the study or could choose not to consent and exit the study. As all participants in the study were over the age of 18 years, consent from their caregivers was not required.

The participation of youth in the study was voluntary, and there was no financial or tangible benefit attached. Youth who were involved in Phase 1 of the study were asked verbally and confirmed in written format if they would like to continue with their involvement in the study during Phase 2 and thereafter during Phase 3.

#### 2.3.2 Description of youth with use AAC

In total, six youth with severe communication disability consented to participate in Phases 1 and 2 of the study and three consented to take part in Phase 3, as shown in Table 1. The age range of the youth was between 19 and 34 years. Three were female and three were male, and all relied on communication support through the use of AAC. Three youth typically used computers with communication software (all three used the Grid), two youth used tablets (iPads) with a communication application (Verbally) and one youth used a cell phone with a communication application (Speech Assistant).

**Table 1:** Description of youth participants who use AAC

Youth	Age	Gender	Regular AAC system	Mechanisms of communication used for participation in the study
Youth 1	34	Female	A laptop computer running the Grid 3, accessed using switches.	1. Completion of an online questionnaire on her laptop.
				2. Provision of questions for the interview in advance so that answers could be preprepared.
				3. Use of verbal yes, no answers.
				4. Assistance from caregiver to confirm answers.
				5. Typing of responses during the online interview using switches for access.
				6. WhatsApp group chat accessed through the computer.
Youth 2	25	Female	A tablet running the Grid 3, accessed through eyegaze.	1. Completion of an online questionnaire on a phone.
				2. Use of verbal yes, no answers during a WhatsApp video call.
				3. Speech‐to‐speech transmission of verbal answers to questions by a frequent communication partner.
				4. WhatsApp group chat accessed through the tablet.
Youth 3	24	Male	Speech assistant on an android phone, direct access	1. Completion of an online questionnaire on a phone.
				2. Use of speech in response to questions with confirmation of understanding obtained by the researcher through the use of yes/no questions.
				3. WhatsApp group chat using direct access.
Youth 4	26	Male	A laptop computer running the Grid 2, direct access.	1. Completion of an online questionnaire on a phone.
				2. Speech‐to‐speech transmission of verbal answers to questions by a frequent communication partner.
				3. Confirmation of understanding from the researcher was obtained using yes/no questions.
				4. WhatsApp group chat using direct access.
Youth 5	25	Male	A tablet running verbally, direct access. Use of writing using pen and paper.	1. Completion of an online questionnaire on a phone.
				2. Use of speech in response to questions with confirmation of understanding obtained by the researcher through the use of yes/no questions.
				3. Use of pen and paper to write longer answers down or draw images for clarification.
				4. WhatsApp group chat using direct access.
Youth 6	33	Female	A tablet running verbally, direct access. Gestures	Questionnaire and interview:
				1. Completion of an online questionnaire on a phone.
				2. Use of speech in response to questions with confirmation of understanding obtained by the researcher through the use of yes/no questions.
				3. WhatsApp group chat using direct access.

#### 2.3.3 Procedures

*Phase 1*: Phase 1 of the study (preparation) involved the conduct of a needs analysis to identify the needs of individuals with communication disabilities concerning COVID‐19. For the needs analysis, a similar process to that used for obtaining consent was used with the youth (i.e., an online questionnaire). The questionnaire was written using simple sentences. Comprehension of the questionnaire was supported with pictures for the concepts discussed. Each question and possible answers were also presented in auditory format. On completion of the questionnaire, an online interview was arranged with the youth to probe their involvement further. The online interview was conducted using either Zoom or WhatsApp video calling depending on the resources available to the youth. The online interview was conducted by a researcher who is experienced in working with individuals who use AAC. Youth were provided with additional time to answer the questions and were given opportunities to provide answers in different formats according to their communication needs.

One youth chose to write his answers and would then show the paper to the researcher so that she could read their answers. Two of the youth would speak their answers, but had mostly unclear speech, so for each sentence, once spoken, the researcher would repeat what was said and the youth would confirm whether they had been correctly understood or not. If they were not understood, they would repeat the sentence and the researcher would confirm understanding word by word until this was achieved. Once they were understood, the interview would continue. A third youth asked for the questions in advance as they took a long time to produce answers. The researcher provided the questions in advance and then joined the youth online, where they presented the prepared answers using a communication device. The final two youth had regular communication partners with them for the interview. Once the researcher asked a question, the youth would provide their answer to their communication partner, who repeated it to the researcher, thus aiding in the comprehension of unclear speech.

*Phase 2*: For Phase 2 of the project, a WhatsApp (a messenger service that also includes call and video call functions) group was set up with all the youth in the study. WhatsApp was the preferred mechanism for communication with the youth, who consented to this format before the establishment of the group. WhatsApp was preferred as it is a commonly used messaging app in South Africa that uses very little data. All the youth were already regular users of WhatsApp. The use of the messaging app allowed for asynchronous group discussion, as well as providing the youth with time to produce answers to the questions posed. During Phase 2, the youth were asked what vocabulary should be provided on communication boards relating to COVID‐19. The researcher proposed some vocabulary as a starting point, and the youth then added to or modified this. Additionally, the youth also offered input on the cultural validity of the symbols to be used on the communication boards. Work on the symbols was conducted by having an artist join the group chat. A proposed symbol was provided and the youth were asked what they thought it meant. The youth were then given the intended meaning and asked what changes needed to be made to achieve the intended meaning. The artist then changed the symbol as suggested and provided the new version to the youth for their confirmation or further changes. The final element of coproduction was the production of short animated videos on topics relating to health and COVID‐19. The videos themselves were produced by students at the University of Pretoria based on the needs analysis from Phase 1 of the study. Once produced, each video was sent to the youth for their feedback, and recommendations for changes.

*Phase 3*: The third phase of the study (implementation) involved the sharing of the resources coproduced during this study as well as the sharing of experiences during the study. All the youth who participated in the study were invited to attend the information sharing via a webinar. Those who indicated that they would like to be involved were included in producing feedback for presentation at the webinar. The feedback was provided in written format to the researcher, who then added audio reading of the information so the webinar attendees could hear and see the information presented. In providing the audio reading, the youth were asked what ‘voice’ (digital voice) they would like to use to represent themselves. The youth who use AAC were then also able to join the webinar discussion using the ‘chat’ feature on the webinar platform. During the webinar, the researcher ensured that when one of the youth was asked a question, he or she was provided with time to answer in the chat section. The researcher then read out this answer for the benefit of all attendees.

## 3 RESULTS

In this section, the results are presented by first describing the youth and then their roles in the three research phases.

### 3.1 Application of the Involvement Matrix in the research project

The researchers applied the Involvement Matrix to map out and guide the planning of how youth with severe communication disabilities would be involved in this project. The Involvement Matrix is flexible in its application. This means that it may not be necessary to apply all five roles of the Involvement Matrix, but rather consider the possible roles that youth could be involved in.^39^ In this study, the roles of youth as (1) listener, (2) advisor, (3) decision‐maker and (4) partner are reported.

**Figure 1:**
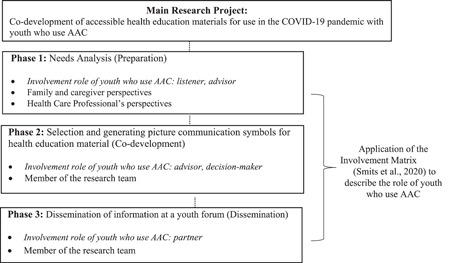
An overview of the main research project. A summary of Phases 1, 2 and 3 of the study is shown. It also shows the application of the Involvement Matrix and describes the involvement role of youth who use AAC in the current study. AAC, augmentative and alternative communication

This process is shown in Figure 1. Procedurally, the researchers planned the phases of the project to overlap with the three research phases outlined in the Involvement Matrix. One member of the research project team led the data collection activities with the youth in Phases 1 and 2. As shown in Figure 1, youth were thoughtfully involved in activities related to the preparation, codevelopment and dissemination to ensure that the intended health education materials were both of interest and relevance to them.

The definitions of the five involvement roles of the youth from the beginning of project to the end are shown in Table 2. Each role will be described in the sections that follow.

**Table 2:** Involvement Matrix roles and role definitions: Current research project

Research phase	Application of Involvement Matrix roles	
	*Youth who use AAC*: Involvement role	Description of involvement of youth who use AAC
Phase 1: Needs analysis	*Listener*: Listens to information	Listened to the information about the research project given by the researcher
Phase 2: Codeveloping health materials	*Advisor*: Gives advice	Advised on the health education topics
	*Advisor*: Gives advice	Advised on the symbols to be included on communication boards as well as the actual structure of the symbols. The youth guided the process of adaptation of symbols for cultural validity, bringing in extra elements for consideration
Phase 3: Copresenting information	*Decision‐maker*: Makes final decisions	Made final decisions on AAC symbols for their communication board
	*Partner*: Works as an equal partner	Youth and a member of the researchers are copresenters of information at a youth forum discussion panel

Abbreviation: AAC, augmentative and alternative communication.

In Phase 1, youth who use AAC served two roles, namely, those of listener and advisor. Phase 1 entailed a needs analysis that was conducted as a semistructured interview with each of the youth through online chats. The researcher (third author) conducted this process using online platforms, that is, Zoom (web‐based video meeting app) and WhatsApp messenger. Video calls allowed the researcher to see the youth with severe disabilities, which helped with understanding the information that the youth provided.

#### 3.1.1 Listener

During the interviews with the youth, the researcher provided information about the aim of the research project verbally and supported the information with visual aids such as pictures. In their role as a listener, the youth listened to the information presented by the researcher. They were allowed to ask questions they had about the project by using their AAC.

#### 3.1.2 Advisor

During the needs analysis, the were youth also involved as advisors. Through the use of AAC, youth advised the researcher on what health education they required during the COVID‐19 pandemic. Their advice was given in response to a set of questions posed by the researcher. An example is shown below:

What makes it easier for you to understand the information that a doctor, nurse, therapist or family is giving you about your health? (Researcher)

(provided his answer by writing his response with pen and paper, and then holding the paper up to the camera during the video call for the researcher to read) (Youth 5)

In turn, the researcher ensured that she clarified her understanding of what the youth had advised by using AAC to support this process:

So did you understand everything? you're happy to carry on with the survey? (Researcher)

‘Yes’ (nodded + vocalized) (Youth)

‘No questions for me?’ (Confirms her understanding of the youth's response by posing a further question on the topic) (Researcher)

‘No’ (head shake) (Youth)

Furthermore, youth advised on their communication challenges in accessing health education during the COVID‐19. For example:

Were there any problems with that (doctors) visit? (Researcher)

Yes, to wait (for) Medical aid. (Youth 1)

Youth also advised the researcher by recommending topics to include in health education materials:

What kinds of words are important for you when you're at the hospital? (Researcher)

A picture with words, because some of us can't point to every place that is sore (Youth 6)

The information and recommendations gathered in Phase 1 assisted the researchers to develop a preliminary set of picture communication symbols for developing health‐related information for use during COVID‐19. This then informed the codevelopment process in Phase 2, where youth were invited as advisors and decision‐makers, as explained in the next section.

#### 3.1.3 Advisor

As advisors, the youth contributed to developing the health education materials by giving their opinion on their choice of vocabulary and AAC symbols on the health topics that they had identified in Phase 1. This process, conducted via WhatsApp messenger, enabled youth to take extra time to provide their opinions.

The researcher presented a range of AAC symbols in the WhatsApp chat to the youth with disabilities and asked their opinions and thoughts on the type, colour and sizes of picture communication symbols. The youth also drew and added images that they thought were better representations of the meanings:

The researcher posted a possible picture symbol on the WhatsApp chat, folowed by asking a question.

Do you like the symbol? It is supposed to mean ‘how will we get there?’ (Researcher)

I don't want to lie I really don't understand them…but I understand the questions (Youth 6)

If you had to draw a picture of the question ‘how will we get there?’

What would you draw? (Researcher)

This is supposed to be someone on a wheelchair and cars, sorry I can't draw (Youth 6)

I like this drawing alot but the person should be on a wheelchair (Youth 2)

The middle is best… there should be a tap and a soap or handwash sanitizer. (Youth 5)

It means there is a paper towel to dry your hands, number 1, nothing to be changed (Youth 1)

The researcher asked the youth what they thought about the colour of the AAC symbols. One youth expressed a strong opinion on the appeal of colour and her feelings about black and white symbols:

I dislike these black and white pictures because I feel like they are too boring for me and can you change the colours and then add some colours that are eye‐catching? (Youth 3)

Other youth had a neutral opinion on the use of colour for the AAC symbols:

For me it does not make a difference (Youth 1)

#### 3.1.4 Decision‐makers

As decision‐makers, youth were given the opportunity to upload their suggestions of what symbols they thought might work. These symbols were either sourced by themselves from the internet or were drawn by themselves. While youth acted mainly in the role of advisors, they also took initiative themselves to make decisions related to age, gender, and race by specifying their preferences, as shown in their responses to various symbols below:

Without colour of a skin (Youth 2)

We mustn't forget about age differences (Youth 6)

Add female symbols because I see male or boy symbols in all of them (Youth 3)

The youth also felt that the AAC symbols should not resemble cartoons or androgynous shapes, but rather depict images of real people:

… they should look like normal people that will be more clear. (Youth 6)

Try to make it look like normal people. (Youth 2)

Throughout Phase 2, as advisors and decision‐makers, the youth enjoyed giving feedback to each other in the WhatsApp messenger group chat:

I think colour because it attracts (Youth 1)

I think the symbols should be in colour, so that it will be easy to identify and I think they convey a message much better. (Youth 5)

For me it does not make a difference. (Youth 6)

I think colour attack (attracts) more people then black and white symbols. The mind of a person remembers colourful pictures than no colour pictures. (Youth 4)

#### 3.1.5 Partner

In Phase 3, three youth were involved as partners in an online youth forum to present the project to a public audience. As important members of the research team, the youth who used AAC partnered with a member of the research team to share the process of the codevelopment process. As partners, the youth presented their thoughts by using their AAC devices. Youth 1 recited a poem that she had written to illustrate the impact of living with a disability during the COVID‐19 pandemic and its impact on mask‐wearing and breathing difficulties. Youth 6 used her speech‐generating device to provide suggestions on how medical and healthcare professionals should be trained on improving their interpersonal skills when interacting with youth with severe communication disabilities during medical consultations.

Members of the audience addressed questions to the youth with severe communication disabilities and as partners of the research team they had an opportunity to answer the questions using their AAC systems.

## 4 DISCUSSION

### 4.1 Overview of findings

Previous literature has highlighted that often health materials, tools, and technologies for youth and children with disabilities are developed by researchers without consideration of the unique role and perspectives of youth.^14^, ^15^, ^16^, ^20^ In alignment with a participatory and inclusive research agenda, we have described one example of an alternative tool that researchers could apply to involve youth with disabilities in research. As such, our findings situate themselves within a greater body of current research focused on applying frameworks and tools to involve youth with disabilities in codeveloping health interventions.^3^, ^15^, ^17^, ^34^, ^41^

This study described the application of the Involvement Matrix in the context of our research project to involve youth with severe communication disabilities in South Africa in the roles of listener, advisor, decision‐maker and partner.^40^ As limited guidelines exist for involving youth with severe communication disabilities in research, this study contributes valuable findings to the growing body of knowledge in this area.^17^, ^45^ In particular, this study expands the research on applying the Involvement Matrix in two significant ways.

First, while this study adds to the application of the Involvement Matrix to youth with chronic health conditions,^3^ it also generates novel insights in supporting the communication of youth with severe disabilities with AAC in its application. Importantly, the implementation of AAC strategies enabled youth with severe disabilities to be involved in the research project more productively and meaningfully. However, this finding raises the criticality of researchers becoming more familiar with using AAC to involve youth with disabilities in research more effectively.^34^ Acknowledgement is made by the researchers that involving youth with multiple disabilities is challenging, especially when using AAC to facilitate their research involvement. Researchers, especially in South Africa, require further training on implementing AAC successfully with youth with severe disabilities.^23^

Second, through the application of the Involvement Matrix,^40^ this paper demonstrates how youth with disabilities in the South African context may be empowered through their involvement in health research during the COVID‐19 pandemic.^35^ In previous studies, researchers have highlighted that to be responsive to the varied lived experiences of youth with disabilities is to enlist their direct involvement in the research process through partnerships with researchers.^34^, ^46^

The researchers of the project were mindful not to rely on their ideas but worked with youth who use AAC to obtain complementary perspectives through their lived experiences as experts of their health condition.^2^, ^40^ The youth embraced the opportunity to be involved in a research project in which their opinions were not only listened to but their advice and decisions were implemented.

The application of the Involvement Matrix in this project is possibly best described through a comment of one of the youth participants to the other youth participants as the final materials were being shared ‘Wow we really did well guys. It's fantastic!’ (Youth 6). The strength of this comment lies in the personal sense of ownership felt by the youth participant in the materials produced. They felt like partners within the process and regarded the work produced as their own and not as work produced ‘for’ them by someone else.

However, the researchers of this project are cautious to suggest such involvement for all youth with severe communication disabilities, as some may not want to be involved in research or may not wish to be involved in all research phases.^2^ Furthermore, some youth with severe disability may experience stress and undue pressure by being involved with researchers in health research.^45^ It is important to ensure the voluntariness of such participation.

This study project contributes to the extant body of literature on how researchers could consider adopting more inclusive research methods with AAC strategies to actively involve youth who use AAC in research.^14^, ^47^ Our findings may suggest that the involvement of youth who use AAC in various roles throughout the research process could possibly lead to outcomes that may potentially be more valued and acceptable to youth.^41^, ^46^

Methodologically, the findings of this project concur with health and disability researchers who have recognized that PPI in research is an important and possibly even a necessary component of a well‐designed research project.^1^ Our study highlights that despite the complexities of severe communication difficulties, involving youth who use AAC in research is possible when it is planned and guided by the roles framed in tools such as the Involvement Matrix.

### 4.2 Strengths and limitations

In this paper, three main strengths emanating from the application of the Involvement Matrix^39^ have been illustrated. First, this project has described how researchers could involve youth who use AAC in the development of health materials by applying the Involvement Matrix^40^ to plan for their roles at different phases of the research process. In particular, where in‐person involvement was not possible due to the COVID‐19 pandemic, synchronous and asynchronous communication technology such as mobile phones and web‐based messenger applications enabled audio and video calls as well as text message exchanges, thereby providing an opportunity to facilitate the involvement of youth with severe disabilities in research. Second, the application of the Involvement Matrix^39^ proposes an example of how disability researchers can begin to think about involving youth with disabilities in research in a systematic way. Third, the paper highlights that even youth who use AAC such as speech‐generating devices and written responses can be involved and can take on different roles throughout the research phases. To the researchers' knowledge, this is the first study to apply the Involvement Matrix^40^ to a research project involving persons who use of AAC.

Two limitations are acknowledged: Technological challenges with internet connectivity may have impacted the process of information‐sharing between the youth and the researcher during Phases 1 and 2 of the study. Additionally, the advent of COVID‐19 may have hampered the recruitment of a broader group of youth, which may have enriched the study findings by potentially facilitating the involvement of a greater number of youths in the research study.

## 5 CONCLUSION

This project has described how youth with severe communication disabilities who use AAC can be involved in health research as listeners, advisors, decision‐makers and partners in the research process through the application of the Involvement Matrix.^40^ Importantly, their involvement could be enhanced when their roles were clear and well defined, and when they could use communication support through AAC. The application of the Involvement Matrix^40^ appears to provide researchers with an opportunity to think clearly and critically about the various roles that youth with disabilities can play and to carefully plan and formalize these roles within an organized framework.

## CONFLICT OF INTERESTS

The authors declare that there are no conflict of interests.

## ETHICS STATEMENT

The research project received ethical approval from the Faculty of Humanities at University of Pretoria (02595761).

## AUTHOR CONTRIBUTIONS

Shakila Dada was the Co‐PI on the SASUF and UNICEF project. She conceptualized the project and contributed to the first draft, data interpretation, writing and revising of the final manuscript. Adele May contributed to the manuscript conceptualization, writing and revising of the final manuscript. Kirsty Bastable was involved in data collection, data analysis, project implementation, manuscript writing and its revision. Alecia Samuels contributed to the writing and revising of the manuscript. Kerstin Tönsing contributed to the writing and revising of the manuscript. Jenny Wilder contributed to the writing and revising of the manuscript and is the Co‐PI on the SASUF funding. Maureen Casey contributed to revising of the manuscript. Constance Ntuli contributed to cowriting aspects of the manuscript. Vasu Reddy was Co‐PI on the UNICEF project and was involved in conceptualizing the project, and revising of the manuscript.

## Data Availability

The data that support the findings of this study are available from the corresponding author upon reasonable request.
